# Integrating ethics in digital mental healthcare technologies: a principle-based empirically grounded roadmap approach

**DOI:** 10.1007/s11019-025-10283-6

**Published:** 2025-07-30

**Authors:** Wanda Spahl, Giovanni Rubeis

**Affiliations:** 1 Division Biomedical and Public Health Ethics, Karl Landsteiner University of Health Sciences, Krems, Austria; 2 Greifswald Medical School, Greifswald, Germany

**Keywords:** Applied ethics, Empirical bioethics, Methodology, Digital health, mHealth, Health apps, Serious games

## Abstract

Digital mental healthcare technologies increasingly incorporate gamification, yet relevant ethical considerations remain underexamined. This paper introduces the *Principle-Based Empirically Grounded Roadmap Approach* (PERA), a methodological contribution to empirical bioethics. It has evolved from ethics research within the Horizon Europe project ASP*belong*, which designs a collaboratively played augmented reality intervention for adolescents. PERA refines existing integrated empirical bioethics methodologies by responding to three key characteristics of the use case: a largely predetermined technology with a relatively low degree of openness in technological design, embedded co-development practices led by facilitators from within the project team, and planned future iterations beyond the ethics team’s involvement. PERA integrates mapping of principles from the ethics literature, a scoping review of the moral intuitions of developers of comparable technologies, and the collection of original empirical data on the use case. Using abductive reasoning, these insights are synthesized into a tangible output: an ethics roadmap designed to guide and be adapted in future use case iterations. By advancing a methodology of combining normative reasoning with empirical insights on a concrete use case, this paper provides both practical tools for ethics researchers in technology projects and a means to generate empirically grounded conceptual contributions. Its outcomes, when brought into dialogue with findings from other integrated empirical bioethics research, can support the critical examination of broader assumptions and implications of gamified mental healthcare, including questions of good care and the broader social implications of such technologies.

## Introduction

Ongoing innovations in digital gamified mental healthcare pose important bioethical questions, with recent reviews highlighting significant research gaps in this area (Damaševičius et al. 2023; Pavarini et al. 2024). Ethics researchers face the challenge of responding to the fast pace of technological development, but there is no unified agreement on methodological standards in contemporary bioethics (Hofmann 2024). Over the past two decades, the so-called “empirical turn in bioethics” (Borry et al. 2005) has promoted integrating empirical research with normative analysis, emphasizing that ethics should not only apply abstract principles to practice but also emerge in response to empirical realities. The turn sparked ongoing debate about how to balance and integrate empirical and normative approaches while preserving the unique strengths of each (Hurst 2010). In response, bioethics scholars have developed different versions of empirical ethics that combine normative theorizing with empirical data analysis (Davies et al. 2015; Molewijk et al. 2004). For example, Ives et al. (2018) defined 14 aspects across six domains that should guide empirical bioethics research, including a practical problem, concrete research questions, integration of empirical and normative analysis, and researcher competence.

Beyond healthcare, scholars in science and technology studies, political science, and innovation research have developed influential interdisciplinary approaches such as ELSI/A (ethical, legal, and social implications/aspects) and Responsible Research and Innovation (RRI). These approaches have emphasized the importance of anticipating and addressing societal and ethical dimensions during technology development. They have shaped empirical approaches to ethics, particularly by promoting user and stakeholder engagement and the integration of ethics researchers into development processes (Hilgartner et al. 2017; Ryan and Blok 2023).

Established methodologies tailored to healthcare technologies include the Ethics Parallel Research and the Embedded Ethics and Social Science (EESS) approach. The former was developed in the context of biomedical technology development, particularly in regenerative medicine and personalized healthcare in the Netherlands (Jongsma and Bredenoord 2020). Ethics Parallel Research aims to systematize ethics research practices that accompany, but are not fully integrated into, technological development. The approach seeks to ensure practical relevance while allowing ethicists to maintain a reflective distance, rather than engaging continuously with developers. The EESS approach, developed in the context of digital healthcare technologies using artificial intelligence (McLennan et al. 2020), integrates ethics researchers into development teams from the outset, emphasizing early collaboration and shared responsibilities (McLennan et al. 2022). It offers useful hands-on tools (Willem et al. 2024) and suggests best practices for ethics research in interdisciplinary technology development, including the establishment of mutual understanding also via key terms and language, the encouragement of active participation, the provision of practical examples and confidentiality with regards to the wider team members’ privacy (Tigard et al. 2023).

**Fig. 1:**
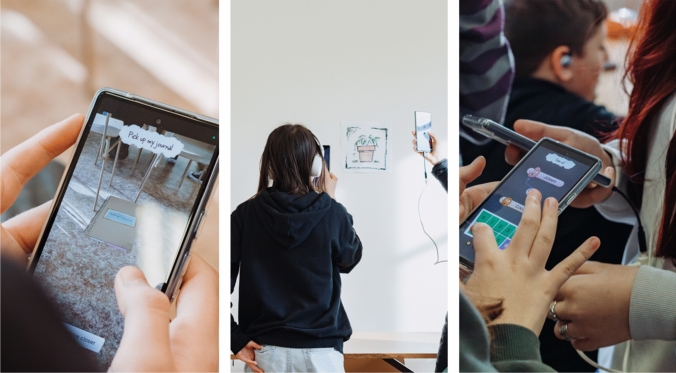
Playtesting of the Lina augmented reality smartphone experience at a Czech school (photo copyright: Evgeniia Tokmakova)

It is against this backdrop that we began our ethics research in the Horizon Europe project ASP*belong*, which develops a new format of gamified digital mental health interventions aimed at young people, called Augmented Social Play (ASP). Based on evidence-based psychology, ASP is delivered via a smartphone app and incorporates augmented reality elements to foster adolescents’ sense of belonging. The first experience, called Lina, is targeted at students and played collaboratively by them in classrooms over 6 weekly sessions (see Fig. 1). Teachers take a leading role in facilitating the intervention, guided by the app and supporting materials, which also include reflective activities beyond the app itself. Its developers span five European countries and bring together a diverse team from within and beyond academia, including game designers, serious games development studio, user experience researchers, computer scientists specialised in artificial intelligence (AI), practitioners facilitating youth engagement, psychology researchers, and a non-governmental organisation (NGO) specialised in youth mental health. Once Lina is complete, a psychology-led trial with young people aged 12–13 in the Czech Republic, Portugal, and the UK will assess its effectiveness in the third year of research.

This paper outlines our methodological approach to conducting ethics research during the development of Lina. We adapt existing integrated empirical bioethics approaches to the specific demands of this concrete use case. The paper addresses the following research question: How can ethics researchers methodologically accompany digital mental healthcare technology development when (1) the technology is largely predetermined, (2) co-development practices are embedded, and (3) future iterations are anticipated?

(i) ASP is a largely predetermined technology, as its first iteration, i.e. Lina, builds on an earlier prototype, which demonstrated high acceptability and preliminary efficacy: a single-session augmented reality smartphone experience for children aged 10–13 (Mittmann et al. 2022). While the core technology remained largely unchanged, Lina adapts this foundation to different countries’ specific contexts and scales it into a multi-session intervention. Because of this predetermined basis, we found that “ex ante” approaches—future-oriented ethics and social science methods often applied to emerging technologies (Reijers et al. 2018)—were not well suited to our case. These methods typically assume a high degree of openness in technological design and focus on shaping trajectories early on, whereas Lina already had a defined structure and direction when our work began.
(ii) Another core characteristic of Lina development is the strong integration of participatory methods, through a variety of co-development practices which involve young people (see Table 1) and other relevant stakeholders such as teachers, psychotherapists and other experts across all project stages. These processes were not initiated by the ethics researchers but were already embedded in the project design and carried out by facilitators from within the project team, including both academic and non-academic members. This distinguishes our setting from many established empirical ethics methodologies, where participatory engagement is typically introduced and managed by the ethics team itself. Instead, our task was to methodologically build on these existing participatory structures and to ensure that ethical reflection could accompany and respond to them appropriately.
(iii) A goal of the larger ASP*belong* project is to initiate the development of future iterations of the technology, with planned adaptations tailored to vulnerable groups. For our ethics research, this meant ensuring that our findings would remain accessible and usable for developers involved in future iterations beyond the duration of our involvement. In contrast to embedded ethics approaches such as EESS, where ethical reflection is often closely tied to the active presence of ethics researchers within development teams (McLennan et al. 2020, 2022), our task also involved developing outputs that could carry ethical considerations forward independently.

**Table 1:** Overview of co-development practices with young people in the ASP*belong* project

Co-development practices	Facilitators	Project development phase
Weekly in-person co-development workshops with a group of “creative associates” (aged 12–14) in the United Kingdom (UK) over 7 months	London-based youth theatre	Year 1
Three in-person international youth exchanges in the Czech Republic, Portugal, and the UK (aged 12–14; see (ASP*belong*^2025^)	London-based youth theatre, psychology researchers from the UK, the Czech Republic, and Portugal	Year 1 and 2
Bi-weekly online meetings with ten “adolescent co-researchers” (aged 14–16 years at project start, from four countries)	Consultation available for all project team members (activities coordinated and led by a Birmingham-based phenomenologist)	Year 1, 2, and 3
Three workshops on interactive AR markers with children (aged 12–13) in the Czech Republic	Brno-based user experience researchers, joined by an external local school psychologist	Year 1 and 2
Testing the alpha and beta version of Lina in schools in the Czech Republic, Portugal, and the UK, including feedback from and observation of adolescents during play	Local psychology researchers	Year 2 and 3

Our ethics research aims to provide practical guidance for developers while generating conceptual insights grounded in the Lina use case. This paper addresses our methodological approach to achieving these goals, which we call PERA—the *Principle-based Empirically Grounded Roadmap Approach*. The acronym also alludes to the Latin term *pera* (satchel), evoking the notion of a portable and adaptable ethical toolkit.^1^ While we build on existing approaches in integrated empirical bioethics (Jongsma and Bredenoord 2020; McLennan et al. 2020, 2022), we expand them by offering a hands-on model for conducting research in a project with a largely predetermined technology and established user and stakeholder involvement, and by introducing an ethics roadmap as a tangible outcome to guide future iterations of the technology.

## The principle-based empirically grounded roadmap approach

The approach described in this paper recognizes that addressing the challenges of emerging technologies requires empirical research and interdisciplinary collaboration between ethicists and developers (Floridi and Taddeo, 2016). It engages in normative bioethics, as defined by Rehmann-Sutter et al. (2012), to ‚contain prescriptive elements that say something about what practitioners should do, why they should do so, what they could reasonably aim at, or how they can reach good decisions." (Ibid., p. 436) Unlike traditional principlism (Beauchamp and Childress 2019) and non-empirical ethics research methods (Ursin et al. 2024), the methodological approach outlined in the rest of this paper is empirically grounded. To meaningfully specify and adapt ethical principles, it draws on empirical insights from a concrete use case, recognising that such principles are not universal but situated within specific local contexts (Wendehorst et al. 2024).

PERA integrates normative and empirical insights from multiple data: It engages in (a) mapping of principles from the ethics literature on similar technologies, (b) a scoping review of the moral intuitions of developers of comparable existing technologies, and (c) the collection of original empirical data on the use case. While (a) follows an understanding of ethics based on normative reasoning, (b) and (c) allow to learn from the practical knowledge and the moral intuitions of game developers. Building on Guillemin and Gillam’s (2004) concept, derived from Komesaroff's (1995) bioethical work, we view “ethics in practice” as the everyday ethical issues that emerge unexpectedly during intervention development. These “ethically important moments” (Guillemin and Gillam 2004) occur when developers must respond spontaneously to situations where ethical harm could arise (Carter et al. 2017). Through empirical insights on how ethical aspects have been navigated in the use case and comparable technologies, PERA captures these moments for ethical analysis.

The outcome of an ethics roadmap harvests these principle-based and empirically grounded insights: Using abductive reasoning, it combines key insights derived from the ethics literature on normative principles and the “ethics in practice” knowledge from the development of the first iteration of ASP, i.e. Lina, and from other existing interventions. In recent years, the philosophical tradition of pragmatism has resurfaced in the form of abduction as approach to data analysis, both in the social science of health and empirical healthcare ethics (Tavory and Timmermans 2014; Timmerman et al. 2019). The strength of abduction lies in its ability to bridge theoretical propositions from normative ethics with empirical insights from a concrete use case, fostering a “continuous dialectical relationship that empirical work has with the theoretical” (Carter 2009). Thereby it is a useful analysis method for empirical bioethics.

### Mapping of ethical principles in the existing literature

Integrated empirical ethics is useful when there is a lack of comprehensive ethical knowledge for a new technology or a specific use case under development. However, ethical issues raised by new healthcare technologies are in most cases not entirely new but rather extensions of existing ethical dilemmas that can be addressed with established theories and concepts (McGinn 2010).

Building on this understanding, we mapped relevant ethical principles from the existing literature, including ethical guidelines, frameworks, analyses, and scoping reviews addressing gamified digital mental health interventions for young people. While no single publication addresses all these aspects, we anticipated that studies such as Wies et al. (2021)—a scoping review on digital mental health for young people without a specific focus on gamification—would still offer valuable insights for our work. The principles identified in the selected ethics literature were organized into a clearly structured overview for further analysis. Each principle was listed with its original formulation, accompanied by a paraphrased summary of the authors’ interpretation.

Unlike ethical analyses that begin with a predefined set of ethical principles as a foundation for evaluation, our mapping draws from multiple sources to avoid such constraints. Instead, it compiles a broad overview of principles that ethics research has identified as relevant for technologies raising similar ethical concerns.

### Scoping review

To explore the “ethics in practice” (Carter et al. 2017; Guillemin and Gillam 2004) in similar serious games like Lina, we conducted a scoping review on ethical aspects addressed in previous publications on the development of gamified digital mental health games for young people (Spahl et al. 2024). Our aim was twofold: to understand how well-established principles, such as privacy, had been meaningfully specified in prior use cases, and to capture the breadth of ethical aspects discussed. To achieve this, we analyzed 38 articles on gamified digital mental health games for young people, applying a broad understanding of ethics and moral intuitions—going beyond aspects explicitly labeled as “ethical” by the authors (for details, see Spahl et al. 2024).

### Original empirical data

To understand the unique characteristics of ASP and the “ethics in practice” (Carter et al. 2017; Guillemin and Gillam 2004) at play in its development, we collected original empirical data on the use case. Empirical bioethics methodologies span a spectrum from consultative to dialogical approaches (Davies et al. 2015). Consultative approaches involve an external ethics researcher gathering insights from participants, analysing data, and developing normative conclusions, either to address specific problems or to advance theory. Dialogical approaches, in contrast, foster shared understanding through dialogue between stakeholders, such as developers and the public, focusing on concrete issues.

In our research, we combined methods based on consultation and methods based on a more dialogical approach. Consultation methods allow us to access the often implicit, work-related practical knowledge of developers, while dialogical methods facilitate discussions of preliminary findings. Data collection began early, in the first development months of the first iteration of ASP, i.e. Lina, with the ethics team integrated into the ASP*belong* team from the project’s outset.

Our focus is not on the quantity of interviews or other data collected but on understanding the development processes deeply enough to identify their ethical dimensions. This required conducting data analysis alongside data collection. Once the first data on Lina development was gathered, the analysis process began. We employed thematic analysis by Braun and Clarke (2006), assigning themes and sub-themes to coded data extracts from transcribed interviews and other data items. Using Atlas.ti software, we inductively coded the data, iteratively refining themes and sub-themes. An exception was participant observation notes. Due to privacy concerns and the ethics researcher’s dual role as team member and observer, we did not systematically extract data from these notes. Instead, we used memos to capture relevant ethical aspects and mitigation strategies, which informed later interviews, ethics workshops, and discussions.

#### Consultation methods

(i) We conducted one-on-one interviews with Lina developers, though some interviewees preferred to be interviewed in pairs, as they worked closely together and wanted to discuss their work as a team. This approach helped gather detailed insights into their often implicit ethical considerations. As integrated ethics researchers we were also colleagues of the interviewees. Accordingly, it was crucial to clarify the research goal through mechanisms like pre-circulated participant information and informed consent. Reassuring interviewees that transcripts would be accessible only to the ethics research team helped foster open discussion and encouraged them to share ethical challenges related to their work and team dynamics.
  The interviews followed a guide with open-ended questions. It contained introductory questions and four thematic blocks (for an overview of the guiding questions, see Table 2). If certain questions were addressed naturally during the conversation, they were not repeated. Interview guides were tailored to each interviewee’s specific tasks (for example, adding a question about the adaptation process when speaking to team members involved in international implementation). While the guide was inspired by existing research on ethics in digital healthcare technologies, serious games, and augmented reality (Ahmadpour et al. 2022; Christopoulos et al. 2021; Garani-Papadatos et al. 2022; Kiran et al. 2015; Sandovar et al. 2016; Wies et al. 2021), these insights were primarily used to actively prompt moral reasoning through targeted follow-up questions and employ counterfactual reasoning (Carter et al. 2019), rather than asking directly about ethical principles.

**Table 2:** Guiding questions for the qualitative interviews with Lina developers

Introductory questions	• Can you describe your position and your role in ASP*belong*? • Can you describe a typical workday when you are working for ASP*belong*?
Thematic block I: work process	• Did you have to make any difficult decisions? • What interests/values were weighed against each other? • What was the decision-making process? • What are you most proud of in this project? What is it about that that makes you proud?
Thematic block II: hopes and motivation	• What motivates your work in ASP*belong*?
Thematic block III: external factors	• What role do research funding from the European Union in the Horizon scheme play for the app? • And larger societal developments such as a “technology hype”?
Thematic block IV: ethical principles	• What would you consider as an ethical issue (ethical principle)? • Is your work guided by specific ethical principles or frameworks?
(ii) As ethics researchers, we had access to extensive project documents, including work package deliverables and steering committee reports outlining progress and challenges. We analysed these as part of our data corpus. When available, we also included data generated from the co-development practices embedded in ASP development, including for example summaries of interviews conducted with psychology experts, and written feedback or drawings created during the co-development workshops of the London-based youth theatre.
(iii) Another data collection method was participant observation during regular project meetings, including in-person consortium gatherings held two to three times per year and monthly online check-ins with the full project team. We also attended smaller meetings as observers. Notes were taken during and after these meetings.

Table 3 summarises practical instructions based on lessons learned from using consultation methods during our research.

**Table 3:** Lessons learned for applying consultation methods

	Method	Practical instructions
Consultation methods	Qualitative interviews with developers	• Collecting valuable input from all contributors to technology development beyond game designers in the narrow sense, including jointly discussing who counts as a “developer” within the project (for example, practitioners facilitating youth engagement were included as developers of the first iteration of the use case after the broader team highlighted their central role, whereas those working on project dissemination were not)
		• Gaining knowledge on ethically relevant • Not possible to pose direct• Ensuring thorough preparation of interviewees prior to the interview (for example, sending a written invitation via email, emphasizing no prior preparation is needed because of the focus on their practical perspective; Circulating participant information and informed consent forms, for clarifying the research context; Clearly communicating confidentiality regarding transcripts to ensure trust and openness)
		questions about ethics when collecting data from non-ethicists (prevailingly narrow understanding of ethics solely as research ethics, as shown in our scoping review on the moral intuitions of developers of existing interventions (Spahl et al. 2024) and in our empirical research (for example, one developer preferred not be interviewed, explaining that a colleague was responsible for submitting the application to the university’s ethics committee))
		parts of their work through a practice focus (for example, introductory question to encourage detailed description of their everyday work practices: “Could you describe a typical workday when you are working for the project?”)
		• Actively prompting mora• Encouraging detailed description through simple follow-up questions ( “Can you elaborate on this?”, “Can you give an example of this?”) l reasoning through targeted follow-up-questions (inquiring into moral experiences and tensions without overwhelming interviewees with ethical concepts, for example asking “Did you have to make any difficult decisions?”, “What motivates your work?”, “How "groundbreaking" is your work? What are you most proud of in this project?”)
		• Keeping existing ethics literature and principles in mind to probe relevant issues and ask counterfactual questions when interviewees described practices ambiguously discussed in ethics literature (for example, asking about privacy considerations when an interviewee described the potential of biomarker or AI technologies, which require the collection of particularly sensitive personal data)
		• Following up on specific ethical aspects the interviewee mentioned earlier (for example, asking about an ethical principle referenced in a previous project meeting; Inquiring into a mentioned aspect, which was considered relevant within the existing ethics literature)
	Analysis of project documents	• Actively collecting documents produced by the development team, which might yield relevant information for ethics research (for example, reports from different work packages, summaries of interviews with consulted psychologists)
	Participant observation during meetings and other events	• Sensitively balancing the role as team member and as ethics researcher (for example, prioritising participation over observation when appropriate; regularly informing team members about ethics research such as when recording an ethics workshop) • Guaranteeing team members’ privacy (for example, not directly quoting a team member from larger meetings without consulting them)

#### Dialogical methods

(iv) Ethics workshops with ASP*belong* team members were held during in-person consortium meetings to jointly engage in in-depth discussions on ethical issues of project development. Instead of presentations on our research progress, we designed them to include meaningful input on ethical questions in order to facilitate a joint discussion, facilitated through group work tasks on specific ethical issues (for details, see Table 4). Moreover, we also conducted ethics workshops with adolescent co-researchers involved in the project (see Table 1) over several sessions, adapted to their needs in terms of language and content.
(v) We facilitated exchanges on our ethics research and specific ethical aspects through two main formats. First, we aligned our ethics research methods and outcomes with the broader project team. An early brainstorming session helped define the scope of our work and clarify responsibilities, establishing what the EESS approach calls an informal “protocol” (McLennan et al. 2022). This protocol evolved over time and was refined in follow-up meetings once or twice a year, where the role and contributions of the ethics researchers were jointly discussed and adjusted to the project's needs. We also regularly shared preliminary findings and draft papers with the entire team and actively communicated key insights to those for whom they were most relevant. In general, this approach was limited by the developers’ workloads and varying levels of interest in ethics research. While some colleagues engaged actively with our work, others showed little involvement, which we accepted.
  Second, we held one-on-one or small group discussions on issues relevant to specific areas of work. A characteristic of Lina development was the diverse team of developers. This also meant that not every ethical aspect was relevant to all developers. For example, when discussing gameplay mechanisms and aesthetics, we ensured the participation of game designers, serious games development studio, and user experience researchers, while practitioners facilitating youth engagement were kept informed but not actively involved in these discussions. Another example is a thematic exchange with the London-based youth theatre team that led co-development activities on the ethics of co-development practices. Although initiated by the theatre, we shared key insights with the broader team during our second ethics workshop with team members. Given the strong integration of co-development practices across the project (see Table 1), we designed a reflection exercise for the whole team. These joint reflections made visible to the team the differing understandings both across academic disciplines and between academic and youth work perspectives.
(vi) We engaged with Lina developers through shared tasks like co-authoring publications, presenting at conference panels and organising workshops at conferences. These collaborations helped translate ethics research into practical terms while deepening our understanding of developers’ needs. For instance, co-authoring our scoping review (Spahl et al. 2024) with interdisciplinary colleagues ensured accessibility for non-ethicists.
(vii) Regular exchanges with other ethics researchers working on digital healthcare technologies outside of the ASP*belong* project were invaluable for discussing principles and refining empirical methods for studying technology development. Engaging in conversations on concrete challenges of integrated bioethics research, particularly with those in similar projects, proved especially fruitful. These exchanges included bilateral collaborations, presentations at ethics conferences, and guest lectures at bioethics institutes.

Table 4 provides a summary of lessons learned from using dialogical methods for ethics research in ASP*belong*.

**Table 4:** Lessons learned for applying dialogical methods

	Method	Practical instructions
Dialogical methods	Ethics workshops with developers	• Respecting developers’ time (for example, scheduling workshops with project team members during larger in-person consortium meetings to maximise the use of their limited time and include as many participants as possible in the discussion; Clearly explain how the workshop activities support their work and the larger project’s goals)
		• Facilitating dialogue so that developers can meaningfully contribute insights and reflect the moral implications of their work in a two-way process (for example, not presupposing knowledge of ethical concepts; Designing workshops around concrete issues or specific principles instead of asking for general feedback; Avoiding jargon and the introduction of implicit theoretical assumptions; Adapting language; Introducing issues and principles tailored to the specific project, such as presenting pseudonymised direct quotes from previous interviews with team members)
		• Create an engaging format that combines input from ethics researchers with active elements for participants (for example, in the first workshop, the team was presented with an overview of ethical aspects relevant to developing digital mental health interventions. They were then asked to choose an area they found particularly relevant to their work and discuss in groups: “Which concrete ethical challenges do you encounter in this area? How do you (plan to) navigate them?”; In the third workshop, developers were invited to write down “what could go wrong” in collaborative play and augmented reality on sticky notes. These notes were then discussed in small groups and used to identify and structure key themes)
		• Ensuring sustainable use of insights (for example, we synthesised the results from the third workshop into a structured map using visually engaging mindmapping software, which also allowed team members to make use of the workshop insights and to contribute further input after the session)
		• Aiming to include the input of those who do not speak up (for example, sensitively moderating the discussion, at best with the help of a second ethics researchers; Active involvement in breaks and other informal occasions, which can prompt quieter persons to share their ideas; Following-up on groups through other methods, who do not engage in the workshop format)
		• Valuing developers’ input through later adopting their ideas or providing explanations for not doing so (for example, following team members’ wish to include certain aspects they consider particularly relevant in the ethics roadmap)
	Ethics workshops with adolescent co-researchers	• See practical instructions for “Ethics workshops with team members” • Adapting workshops to young people’s needs (for example, using non-academic language; Offering interactive presentations; Regularly checking in to ensure they can follow along) • Preparing the workshop in consultation with someone experienced in working with young people (ideally someone who already knows the group)
	Exchanges on specific ethical aspects	• Facilitating participation by proactive coordination with project leaders and targeted invitations • Respecting developers' time by focusing only on the ethical aspects for which the exchange was organized • Remaining open to developers' needs (for example, allowing them to discuss issues outside the meeting’s primary goal; Supporting them in one of their tasks when reasonable and compatible with the ethics researchers’ general workload)
	Joint publications and conference participations	• Finding a work mode (for example, openly communicating about what the ethics researcher can contribute at the beginning of the collaboration; Clearly defining concepts) • Thoughtful consideration of the ethics researcher's resources (ideally, identifying panels, publications, etc., which also support the larger ethics research)
	Discussions with other ethics researchers	• Exchanging insights with the community of ethics researchers working on digital healthcare technologies (related to content, methods, and concrete challenges)

### Constructing the ethics roadmap

**Fig. 2:**
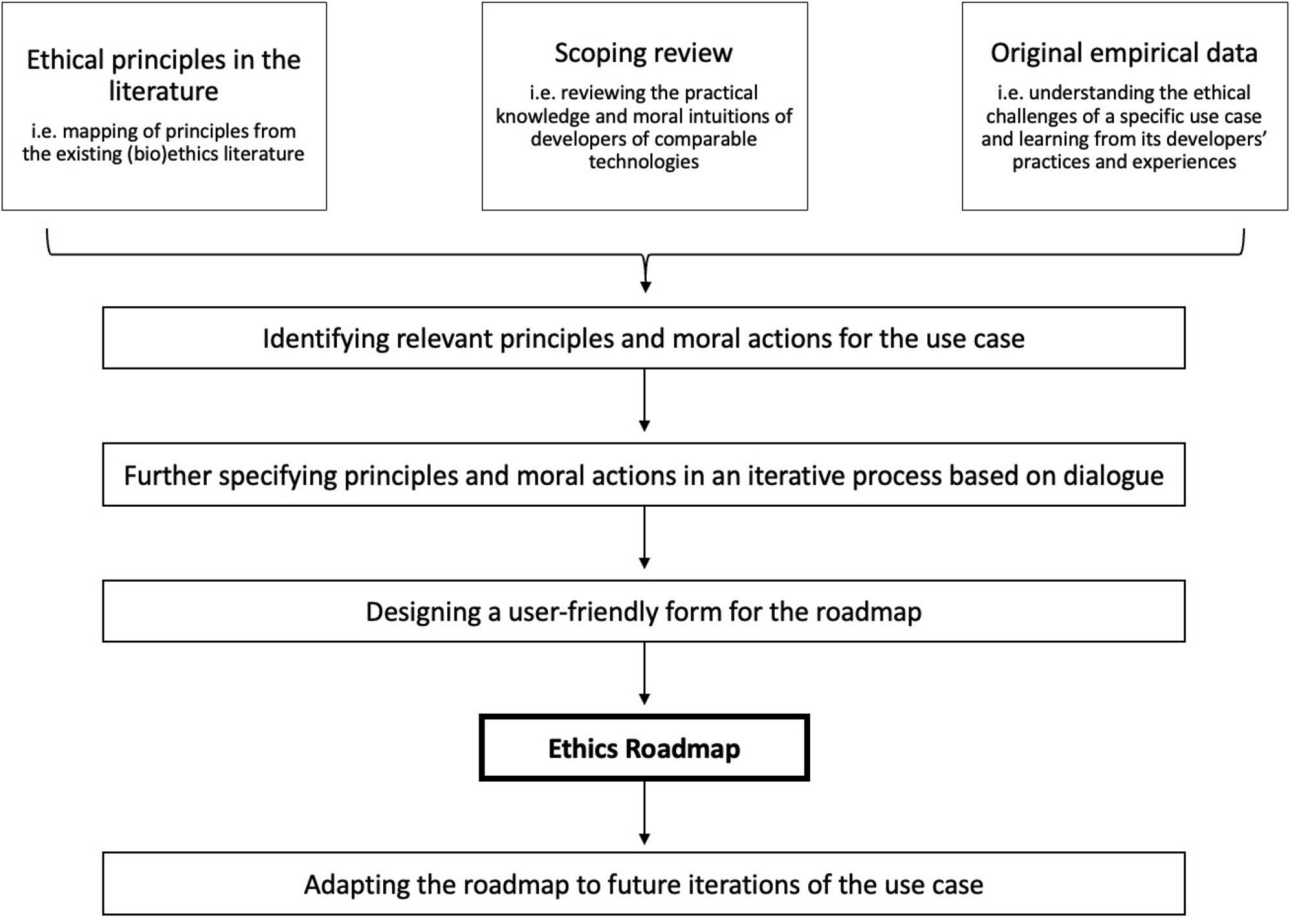
Work steps for creating the ethics roadmap

Creating the ethics roadmap follows several simultaneous and interrelated work steps (for an overview, see Fig. 2). First, relevant principles and moral action instructions in the form of hands-on good practice examples are identified through an abductive approach (Tavory and Timmermans 2014; Timmerman et al. 2019), rooted in pragmatism. This approach emphasizes the dynamic interplay between empirical insights and theoretical propositions that continually inform and reshape one another—where theory enables us to “see things in the empirical that we would gloss over” (Tavory and Timmermans 2014, p. 2), while empirical insights, in turn, redirect and challenge theoretical presuppositions.

In the analytical process of bringing together the principle-based insights from the literature and the empirically grounded findings from the scoping review and the empirical data, we have iteratively refined ethical principles by aligning them with empirical findings from the scoping review and the original empirical research on Lina development. This process means tacking back and forth between the former and the latter in two directions. In one direction, we applied ethical principles from the existing ethics literature by adapting their meanings to our real-world use case. This involved specifying broad definitions through addressing use case-specific challenges and providing practice-relevant examples. For instance, we redefined “accessibility” to include considerations relevant to the context of augmented reality interventions, such as the practical adjustment of placing AR markers within reach of all players, including wheelchair users.

In the other direction, empirical insights guided the selection, refinement, and organization of ethical principles in the roadmap, helping determine which to include, which to omit, and how to organize the roadmap. For example, Lina’s collaborative design provides clear instructions to players, such as directing them to find another player using a specific symbol displayed on their phone. While these gameplay mechanisms could be critiqued from a perspective for restricting autonomous decision-making, they actively promote social interactions. Acknowledging this, we incorporated the principle of “social connection” into the roadmap—an aspect that had been discussed in only one of the papers identified in our mapping of the existing ethics literature (i.e., in Pavarini et al. 2024). This highlights how in-depth engagement with the use case helps determine which ethical principles are most relevant for a given technology. Rather than preselecting a fixed set of ethical principles at the outset of the ethical analysis, PERA maps principles from various sources and assesses their relevance through an iterative dialogue with the use case, possibly including the introduction of new principles.

Second, the ethics roadmap is constructed in an iterative process based on dialogue, in which identified principles and moral actions are specified further and revised. This involves exchange within the ethics team and with academic colleagues in ethics and the social sciences. Our use of dialogical methods (for details, see above) fostered open discussions and opportunities for iterative feedback on preliminary findings from the use case developers, aligning with our abductive approach to data analysis, which proposes to assess insights through dialogue with the “community of inquiry” (Timmerman et al. 2019). We understand this refinement of the ethics roadmap as an ongoing process with no definitive endpoint—similar to the ongoing construction of a transportation network, which is continuously adapted to changing conditions and new mobility needs.

Third, the form of the ethics roadmap requires careful consideration, so that it can support a diverse group of developers (i.e. adolescents, arts sector, technology industry, psychology researchers, engineering researchers) in the future without the guidance of ethics re searchers. We aimed at circumventing the “PDF-ization of ethics” (Goñi et al. 2024), i.e. the dissemination of PDF documents with ethics recommendations and moral action instructions that are difficult for non-ethicists to comprehend and apply in their work. Instead, our ethics roadmap will be hosted on a website with interactive elements, enabling users to explore the content for themselves. Particular emphasis is placed on accessible language, concise descriptions, and an appealing aesthetic. These aspects were developed in collaboration with a user experience designer using the collaborative, web-based design tool Figma.

Fourth, the metaphor of a roadmap inherently conveys adaptability—much like a real roadmap that can be updated to reflect newly constructed roads, route changes, or shifting navigation priorities. It expresses the idea that ethics in emerging digital mental healthcare technologies is not static but should evolve in response to new insights and in dialogue with technological developments in practice. The roadmap embraces conceptual openness, allowing developers to modify it for their own purposes and to address the empirical particularities of their specific use case. This is supported by a non-hierarchical structure of principles and numerous examples designed to encourage reflection in light of one's own project. Additionally, the Figma design is made available for adaptation.

## Discussion

As ethics researchers engaged in a research and development project focused on creating a new format of gamified digital mental health interventions for young people, it was crucial to continuously articulate and adjust our role and responsibilities, as well as how developers contributed to the ethics research. Building and maintaining a trusting, reciprocal relationship between the ethics team and the development team is crucial from the start, as the quality of empirical ethics research depends heavily on *how* ethics researchers collaborate with developers beyond mere procedures (Balmer et al. 2016; Tigard et al. 2023). Bietti (2020) identifies two reasons for “ethics bashing” in technology development: misunderstandings that frame ethics as either profit-driven “ethics washing” or irrelevant moralizing. In response, Bietti calls for an aspirational approach to ethics. Countering this, Bietti advocates for embracing ethics as an aspirational practice.

Taking this seriously, we clarified our role early on—not as watchdogs restricting development, nor as lapdogs legitimizing it (Baker 2005; Bietti 2020). Instead, we explained how we understand ethics as a collaborative process, not aimed at prescribing “right actions”, but at “learning from each others’ pre-existing ethical knowledges”, as we phrased on our slides for the first ethics workshop. As our research began, we were happy to find developers to be highly engaged and enthusiastic about broader ethical questions during the workshops and other interactions, despite their recurring descriptions of institutionalized research ethics procedures as tedious. For example, qualitative interviews with team members often exceeded the planned duration and were remembered as meaningful opportunities for reflection.

In response to our overarching research question—how ethics researchers can methodologically accompany digital mental healthcare technology development when (1) the technology is largely predetermined, (2) co-development practices are embedded, and (3) future iterations are anticipated—PERA offers a context-sensitive and adaptable contribution. Addressing part (1), it engages with ethical issues that emerge even after central design decisions have been made. In the Lina use case, core elements such as the use of collaborative AR game elements and the concept of good care were already defined. Rather than seeing this as a limitation, we treated it as a common feature of applied research and responded by working reflexively within these constraints. For instance, we integrated examples and mitigation strategies into the ethics roadmap, knowing not all could be implemented within Lina. In dialogue with developers, we agreed to treat the roadmap not as a checklist, but as a tool for informed decision-making and ongoing reflection. This demonstrates that ethics researchers can meaningfully contribute to technology development even without early involvement, by helping articulate ethical tensions and providing structured tools for continued ethical engagement.

In the following, we further contextualise PERA within the broader methodological discussion in empirical bioethics—addressing parts (2) and (3) of the research question– and outline limitations and future routes for research.

### Contextualisation in empirical bioethics

PERA is grounded in the tradition of integrated empirical bioethics, which emphasizes situated ethical reflection informed by empirical insights. It aligns with existing empirical ethics approaches, in particular with the EESS approach (McLennan et al. 2020, 2022), and “ethics parallel research” (Jongsma and Bredenoord 2020) in its commitment to integrating ethical inquiry into ongoing healthcare technology development.

We place emphasis on the intrinsic value of the work-in-progress of ethics researchers in technology development projects. The different methods employed in our integrated research fostered reflection among developers. For example, many valued the interviews, some noted that the scoping review broadened their understanding of ethics beyond research ethics, and others shared that the ethics workshops prompted them to think about aspects of their work they had never considered before. Thereby, ethics researchers play a crucial role in helping developers recognize and activate their often-implicit knowledge of ethical aspects, which they have acquired through their everyday practices. Introducing the distinction between formal ethics frameworks and “ethics in practice” (Carter et al. 2017; Guillemin and Gillam 2004) to the larger team proved especially helpful in this regard.

By opening up diverse possibilities for fostering spaces of reflection, the approach described in this paper aligns well with the EESS approach and its practical toolbox (Willem et al. 2024). In terms of the ethics researchers’ role, however, our work is more closely aligned with the Ethics Parallel Research (Jongsma and Bredenoord 2020), which emphasizes accompaniment rather than full integration. This was partly due to the predetermined nature of the technology and partly shaped by the geographically distributed nature of the international team, which at times made close collaboration more challenging.

PERA offers a distinctive contribution by diverging from existing integrated empirical bioethics methodologies in two key ways. First, in addressing part (2) of the research question, our approach adapts to the specific context of already embedded co-development practices by engaging not directly with users and stakeholders themselves, but with those facilitating these engagements. This shift was shaped by the structure of the larger Horizon Europe project, in which co-development with young people and relevant stakeholders, such as teachers, is integrated at all stages of development. While other integrated empirical ethics approaches often engage users and the public more directly, our approach was shaped by the existing structures of involvement. This makes it particularly well-suited to contexts where co-development is an established component of technology design, rather than something ethics researchers must initiate or justify.

Second, in response to part (3) of the research question, PERA produces a concrete outcome: an ethics roadmap. This roadmap is created through a structured, iterative process that synthesizes insights from the existing ethics literature, findings on the moral intuitions of developers of similar digital mental healthcare technologies, and empirical data from the ASP use case. Other approaches often emphasize fostering reflection or embedding ethicists within projects without necessarily aiming to synthesise findings into a formal output. For example, the EESS highlights the value of ethicists being embedded in projects as an end in itself (McLennan et al. 2020, 2022). In contrast, PERA translates situated ethical insights and dialogue with the wider project team into a durable, reflective tool that can guide future development without requiring ongoing involvement from ethics researchers.

### Limitations and future research

The approach outlined in this paper involves specific limitations, while also pointing to fruitful directions for future research in empirical bioethics. First, as an empirically grounded methodology, PERA avoids the risk of imposing assumptions by deducing ethical considerations from abstract principles in a top-down manner. It aims to inspire empirical bioethics research that goes beyond predefined ethics understandings. In the larger Horizon Europe project, team members often struggled to identify ethical issues outside the scope of formal research ethics, even though they were navigating a plethora of ethical issues in their daily work. We created formats, such as the ethics workshops, that support inductive insights from developers’ lived experiences of “ethics in practice” (Carter et al. 2017; Guillemin and Gillam 2004). This points to a broader potential for ethics research: using empirical work not just to confirm existing ideas, but to generate new, practice-based conceptual insights. Conceptually, predefined ethics understandings can limit the ethics analysis to what is already assumed, missing the opportunity to use empirical work to generate new knowledge. Practically, it may also disengage developers, as seen in checklist-based approaches (Green 2021).

Second, PERA was developed in the context of a specific type of digital healthcare technology, namely a gamified intervention targeting youth mental health. This focus has rarely been addressed in ethics research (Damaševičius et al. 2023; Pavarini et al. 2024). Both the approach and its outcome, the ethics roadmap, are tailored to this particular setting. Future studies could further examine the approach’s applicability and value in this emerging field. The ethics roadmap, grounded in empirical findings and specific use case examples, has limited generalizability. Like any roadmap, it offers a structured overview but necessarily simplifies the complexities of real-world development processes. It is not intended as a prescriptive guide, nor does it capture all contextual nuances or provide concrete solutions for every use case. Rather, we understand it as a flexible tool for navigating the ethical landscape of digital healthcare technologies in this setting, leaving room for adaptation and contextual judgement by those applying it.

Third, the roadmap’s conceptual openness and intended adaptability pose practical challenges for implementation. The general insecurity about ethics among some ASP*belong* team members underscored our task to helping surface and articulate these issues. To support future adaptations of the roadmap without continued ethics researcher involvement, future work will need to find ways of making this facilitation process more explicit. For now, our continuing involvement in near future ASP iterations beyond Lina will allow us to both evaluate the outcome of PERA and generate further insights into how the roadmap can function as a dynamic and adaptable tool.

Finally, the predetermined design of the use case we have accompanied meant that ethical considerations began after key design decisions have already been made. For instance, the approach largely adopts the use case’s existing concept of good care, even though this is a crucial topic for discussion before further developing digital mental healthcare technologies (Kleinman and Gardner 2023; Mucić and Hilty 2024). Similarly, it does not fully address the socio-economic context in which these technologies are embedded (Lupton 2018). In future research, more conceptually open “ex ante” methodologies (Reijers et al. 2018) should explore whether and how gamified digital health interventions for young adults align with the concept of good care, how their development is embedded in and shaped by larger socio-economic factors, and what other modes of promoting mental health they preclude.

## Conclusion

PERA offers ethics researchers a methodology for integrating normative principles with empirical insights to generate ethical arguments. It is especially relevant for ethics researchers accompanying projects where a digital healthcare technology is largely predetermined, where co-development practices involve users and stakeholders are embedded, and where development into future iterations continues beyond their involvement. It provides both conceptual insights and practical guidance, notably through the collaboratively developed ethics roadmap—an adaptive and accessible tool that can support development even after ethicists are no longer directly involved. This is particularly important as financial and political investments in digitized, gamified mental health technologies for young people continue to grow, yet explicit ethical reflection remains largely absent as we showed elsewhere (Spahl et al. 2024). The roadmap is tailored to the specific needs of our use case Lina, its future iterations, and similar gamified digital mental health interventions. Yet, we propose PERA as a transferrable integrated empirical bioethics approach for researching and supporting emerging digital mental healthcare technologies more broadly.

While we emphasize the need for tailored and relatable ethics input when developing specific mental healthcare technologies, such as the ethics roadmap for ASP, we caution against reducing ethics research to a mere safeguarding role. Beyond preventing harm and ensuring inclusive gameplay within the scope of a specific use case, ethics research should also critically engage with the underlying assumptions and broader implications of digital gamified mental healthcare. It remains crucial to continue productively questioning what kinds of mental healthcare gamification are beneficial within which contexts, especially considering the broader social implications of prioritizing resources in this direction. For instance, the impact of these technologies on clinical practice and the therapeutic relationship remains a vital area of research, requiring methodologies beyond those discussed in this paper. Likewise, exploring the complex relationships between neoliberal governance and gamification—such as the potential conflict between quality care and financial incentives for digital solutions, or the reinforcement of competitive subjectivity—demands analytical tools beyond those advanced in this paper. However, we view our conceptual insights grounded in the concrete use case of ASP, along with similar findings from other integrated empirical bioethics research, as foundational groundwork for addressing these broader questions in an empirically grounded, informed manner.

## Footnotes

1 The acronym PERA has also been used in unrelated fields, for example, the Purdue Enterprise Reference Architecture in industrial systems engineering and the European Food Safety Authority’s PERA roadmap in environmental risk assessment. Its use here is distinct and specific to the field of digital health ethics.
